# Supplementary material for: Discovery and application of insertion-deletion (INDEL) polymorphisms for QTL mapping of early life-history traits in Atlantic salmon
Source: BMC Genomics. 2010 Mar 8;11:156. doi: 10.1186/1471-2164-11-156 (PMC2838853; doi:10.1186/1471-2164-11-156)
Supplement: Additional file 2 — Information on developed 76 locus single-run INDEL panel in Atlantic salmon. Information on fluorescence labeling, primer concentrations, PCR pooling and links to alignments, INDEL motifs and GENESCAN (Burge and Karlin 1997) predictions of genes/exons are available in html format. [file 1471-2164-11-156-S2.ZIP › Additionalfile2/snpsummary21745.html]

```
Cluster 12182 Contig 1

prev  Summary    Contig List  next
```

Size of Consensus sequence = 786

Number of sequences = 4

Minimum redundancy = 1

Key

A gi|117476484|gb|EG808703.1|EG808703 EST\_ssal\_evd\_29152 ssalevd thymus Salmo salar cDNA Salmo salar cDNA clone ssal\_evd\_538\_079\_rev 5', mRNA sequence  
B gi|117476483|gb|EG808702.1|EG808702 EST\_ssal\_evd\_29151 ssalevd thymus Salmo salar cDNA Salmo salar cDNA clone ssal\_evd\_538\_079\_fwd 3', mRNA sequence  
C gi|117485378|gb|EG817595.1|EG817595 EST\_ssal\_evd\_19815 ssalevd thymus Salmo salar cDNA Salmo salar cDNA clone ssal\_evd\_525\_189\_rev 5', mRNA sequence  
D gi|117485379|gb|EG817596.1|EG817596 EST\_ssal\_evd\_19816 ssalevd thymus Salmo salar cDNA Salmo salar cDNA clone ssal\_evd\_525\_189\_fwd 3', mRNA sequence

6 SNPs detected

A B C D  cosegregation weighted

279 T . G G   6/6 75.00
595 - - C C   6/6 100.00
596 - - G G   6/6 100.00
597 - - C C   6/6 100.00
671 T C C C   6/6 100.00
758 . T A A   6/6 75.00
